# Caractéristiques bactériologiques des infections de liquide de dialyse péritonéale

**DOI:** 10.11604/pamj.2015.22.162.7741

**Published:** 2015-10-20

**Authors:** Manel Jellouli, Meriem Ferjani, Kamel Abidi, Yosra Hammi, Taieb Ben Abdallah, Tahar Gargah

**Affiliations:** 1 Service de Pédiatrie, Hôpital Charles Nicolle, Tunis, Tunisie; 2 Service de Médecine Interne et Néphrologie, Hôpital Charles Nicolle, Tunis, Tunisie

**Keywords:** Péritonite, antibiotiques, dialyse péritonéale, bactéries, enfant, Peritonitis, antibiotics, peritoneal dialysis, bacteria, infant

## Abstract

La péritonite infectieuse (PI) est la principale complication de la dialyse péritonéale (DP). L'objectif de notre travail était de déterminer l’écologie bactérienne des PI et d'adapter l'antibiothérapie selon les germes isolés et les résistances observées. Étude rétrospective effectuée chez tous les enfants traités par DP et ayant présenté une PI dans le service de pédiatrie de l'hôpital Charles Nicolle de Tunis (2004-2013). Au total, 61 ont développé 97 PI. L'incidence des PI était de 0,75 épisode/patient-année. La culture du LDP était négative dans 40 cas. Les Gram Positif ont été notés dans 56% des cas avec prédominance du Staphylococcus aureus. Les Gram négatif étaient retrouvés en seconde position (40%) représentés principalement par le Klebsiella pneumoniae et le Pseudomonas aeruginosa. Des souches de Staphylocoque méticilline résistant étaient isolées dans 21,4%. Les bactéries à Gram positif étaient résistantes aux céphalosporines de première génération dans 25% des cas et aucune résistance à la vancomycine n'avait été décelée. Les bactéries à Gram négatif avaient une résistance globale de 38% avec des souches β-lactamase à spectre élargi (BLSE). L'antibiothérapie empirique devra couvrir les germes à Gram positif par la vancomycine et les germes à Gram négatif par la ceftazidime.

## Introduction

La dialyse péritonéale (DP) est la modalité de dialyse la plus fréquemment utilisée dans la prise en charge des enfants atteints d'insuffisance rénale terminale (IRT). C'est une technique facile, qui préserve la fonction rénale résiduelle et permet de maintenir une croissance acceptable en attente de la greffe rénale [1]. Malgré ces innombrables avantages, la DP présente un risque majeur: la péritonite infectieuse (PI). Elle est redoutable car source de morbidité et de mortalité non négligeables [2]. Elle est également la cause principale de l’échec définitif de cette modalité d’épuration extra-rénale imposant le transfert en hémodialyse, technique lourde et contraignante [3, 4]. La PI peut parfois même engendrer le décès du patient. Les différentes études ont montré la prédominance des infections à des bactéries à Gram positif et en particulier au Staphylococoque à coagulase négative [5]. Les bactéries à Gram négatif représentent 25% des germes isolées [6]. Toutefois, d'autres germes peuvent être isolés plus rarement comme les mycobactéries atypiques, les champignons. L'objectif de notre étude était de décrire à partir de notre expérience sur 85 enfants traités par DP la bactériologie ainsi que les résistances aux antibiotiques afin d'adapter notre stratégie thérapeutique à l’écologie bactérienne retrouvée lors des infections de liquide de dialyse péritonéale dans notre établissement.

## Méthodes

Il s'agissait d'une étude rétrospective transversale descriptive réalisée au service de pédiatrie de l'hôpital universitaire Charles Nicolle de Tunis (1^er^ janvier 2004-31 décembre 2013). Nous avons inclus tous les patients ayant une IRT traités par DP ayant eu au moins un épisode de PI. La PI était définie par la présence d'au moins deux des critères suivants: liquide de dialyse péritonéal (LDP) trouble ou douleurs abdominales, LDP contenant plus de 100 éléments blancs par mm^3^ avec plus de 50% de polynucléaires neutrophiles et culture bactériologique du LDP positive avec mise en évidence d'un germe [7]. Les PI ont été traitées par une antibiothérapie empirique visant à la fois les germes Gram positif et les Gram négatif: il s'agissait d'une association d'une céphalosporine de 3^ème^ génération (ceftazidime 100 mg/kg le premier jour puis 30 mg/kg/j pendant 15 jours), d'un glycopeptide (vancomycine 10 mg/kg/semaine ou teicoplanine 10 mg/kg deux doses le premier jour puis une seule dose par jour, pendant 15 jours); administrés par voie intraveineuse; à un aminoside administré par voie intrapéritonéale (amikacine: 20 mg/poche de 2 litres pendant 3 à 5 jours).

## Résultats

Au total, 85 patients ont été suivis dans notre centre dont 61 ont développé une ou plusieurs PI (72%). Il s'agissait de 35 garçons et 26 filles (Sex-ratio = 1,35). Leur durée totale moyenne de DP était de 20,4 ± 17,1 mois (3,5 - 75 mois). Leur âge moyen de début de la DP était de 8,9 ± 5,6 années (29 jours - 18 ans). Les uropathies malformatives étaient les étiologies prédominantes de l'insuffisance rénale terminale (44%). L'incidence des PI était de 0,75 épisode/patient-année. La culture du LDP était négative dans 40 cas. La bactérie la plus isolée était le *Staphylococcus aureus* suivi des *Staphylocoques* à coagulase négative; avec le *Staphylocoque epidemidis* en première position. Les bactéries à Gram négatif étaient retrouvées en seconde position représentés principalement par le *Klebsiella pneumoniae* et le *Pseudomonas aeruginosa* comme le montre le Tableau 1. Une culture poly microbienne à deux Gram négatif n'a été observée que chez un seul patient. Une origine fongique n'a été constatée que dans un seul cas. L’étude du profil des Gram positif a montré une sensibilité de 100% à la vancomycine et à la tobramycine. Les bactéries à Gram positif étaient résistantes à l'oxacilline dans 16,1% des cas. Vingt-deux pour cent (22,6%) de résistances ont été observées avec la céfalotine. Aucune résistance à la vancomycine n'a été observée parmi les Gram positif. Des souches de *Staphylococcu* sméticilline résistant étaient isolées dans 21,4%. Les Gram positif étaient résistants aux céphalosporines de première générationdans 25% des cas. Les Gram négatif isolés avaient une résistance globale de 38% secondaire à des souches β-lactamase à spectre élargi. Les tests avec la ceftazidime avaient trouvé des Gram négatif sensibles dans 62% des cas, intermédiaires dans 15% des cas et résistants dans 23% des cas; et les tests à l'amikacine 7,1% de Gram négatif résistants. L'antibiothérapie initiale a été maintenue dans 61 épisodes soit 63% et adaptée selon l'antibiogramme dans 36 épisodes soit 37% des cas. Dans la PI à culture poly microbienne l'antibiothérapie initiale a été changée par de l'imipenème selon les résistances cumulées des deux germes isolés. La durée totale moyenne de l'antibiothérapie était de 16,2 ±3,6 jours avec une bonne réponse dans 58% des épisodes. Les complications rapportées étaient: récidive (5%), récurrence (6,5%), hémodialyse temporaire (10%) et hémodialyse définitive (16,4%). L'arrêt définitif de la DP avait été indiqué 66% des patients et était imputé aux épisodes de PI dans 19,7% des cas.

**Tableau 1:** Répartition des différents germes isolés sur le liquide de dialyse péritonéale

Germe		Nombre	Pourcentage (%)
**Gram Positif**	Staphylococcus aureus	20	20,6
	Staphylocoque epidermidis	7	7,2
	Enterococcus faecalis	2	2,1
	Staphylococcus hominis	2	2,1
	Gemella morbillorum	1	1
	Staphylococcus lugdunensis	1	1
**Gram Négatif**	Klebsiella pneumoniae	5	5,2
	Pseudomonas aeruginosa	5	5,2
	Acinetobacter baumanii	4	4,1
	Enterobacter aerugenes	4	4,1
	Klebsiella oxytoca	2	2,1
	Echerichia coli	1	1
	Enterobacter cloacae	1	1
	Serratia odorifera	1	1
Fungique	Candida albicans	1	1
Culture négative		40	41,3

## Discussion

Nos résultats rejoignent les différentes études qui ont révélé que 50 à 60% des épisodes de PI ont été causés par des bactéries Gram positif, 20 à 30% par des germes Gram négatif, environ 2% par des champignons [8]. Les différentes études sont regroupées dans le Tableau 2. Les variations du spectre des bactéries entre les différents centres sont probablement multifactorielles et peuvent inclure les influences environnementales (climat et humidité), l’âge des patients (les plus jeunes sont les plus atteints par les Gram négatif), les divers aspects relatifs au choix et à la configuration du cathéter, aux soins de l'orifice de sortie du cathéter et à l'utilisation de la prophylaxie par une antibiothérapie locale [9, 10]. Dans notre série, les Gram positif étaient les germes les plus incriminés et le *Pseudomonas* était l'un des bactéries à Gram négatif les plus isolés; ceci rejoint l’étude coréenne [11]. Pour le Pseudomonas, l'infection de l'orifice de sortie du cathéter était retrouvée dans 50% des cas dans les autres études contre seulement 20% dans notre série, en raison de la prépondérance des Gram positif [12]. Selon le registre international de dialyse péritonéale pédiatrique (IPPR), le *Pseudomonas* est plus fréquent dans les pays ou les soins de l'orifice du cathéter se font plus que deux fois par semaine, utilisant des agents de nettoyage non stériles pour la plaie et appliquant la mupirocine au niveau du site [9]. L'orifice du cathéter doit donc toujours être gardé sec et propre vu l’émergence du Pseudomonas dans notre série (5,2%), comme dans certains pays subtropicaux, et qui parait être due à l'humidité. Conformément à nos résultats, les champignons ne représentaient qu'une minorité des épisodes de PI avec seulement 2% des épisodes dans le dernier rapport de l'IPPR [13] et 3,1% dans le rapport de la "North American Pediatric Renal Trials and Collaborative Studies" (NAPRTCS) [3]. Les levures appartenant au genre Candida sont les plus couramment impliquées [14–16]. Les facteurs favorisants une PI fungique sont les déficits immunitaires, les antécédents de PI à Gram négatif, l'utilisation préalable d'une antibiothérapie, l'infection de l'orifice du cathéter et la gastrostomie de façon controversée [17]. Warady et al n'ont eux retrouvé aucun facteur favorisant chez 50% de leur patients atteints de PI fungiques [18]. Le *Mycobacterium tuberculosis* est une cause exceptionnelle de PI et n'a pas été retrouvé chez nos patients malgré que notre pays soit un pays endémique pour la tuberculose.

**Tableau 2:** Culture du liquide de dialyse péritonéale dans la littérature

Germe	USA [9]	Mexique [21]	Argentine [9]	Europe De L'Est [22]	Europe De l'Ouest [22]	Turquie [2]	Asie [23]	Notre série
**Fungique**	3	0	0	1	4	2	0	1
**SCN**	11	1	1	16	32	23	1	10
**SA**	10	4	5	6	34	19	2	20
**Streptocoque**	7	1	0	3	13	5	2	0
**Entérocoque**	1	0	0	2	8	9	0	2
**Autres Gram positif**	8	0	1	2	4	5	1	1
**Pseudomonas**	12	0	3	1	2	7	3	5
**Autres Gram négatif**	22	3	13	11	28	26	2	18

**SCN :** Staphylocoque à coagulase négative ; **SA :** Staphylococcus aureus

La fréquence élevée des cultures négatives dans notre série est l'un des points faibles qu'il faut souligner. En Mexique, en Turquie et en Asie, le taux de culture négative variait de 36 à 67% alors qu'il ne dépassait pas les 11 à 23% en Amérique, en Argentine et en Europe [9]. Les analyses de l'IPPR n'ont pas montré de différences dans les techniques de culture [9]. Les causes étaient probablement une antibiothérapie préalable, une erreur du prélèvement, de l'acheminement ou dans le traitement du dialysat recueilli au niveau du laboratoire. L’étude de la sensibilité de nos germes aux antibiotiques n'a pas mis en évidence d'augmentation de leur résistance, ceci laisse supposer qu'il s'agissait dans la plupart du temps d'infections à germes communautaires. La majorité des souches isolées présentaient un profil de sensibilité aux antibiotiques correspondant à des souches sauvages: 79% des Staphylocoques étaient sensibles à la méticilline, aucun *Pseudomonas* n’était résistant à la ceftazidime et il n'a pas été isolé de souches multi résistantes parmi les bactéries Gram négatif. Pour les Gram positif, toutes les souches étaient sensibles à la vancomycine ce qui concorde avec les études précédentes [11], mais beaucoup de souches étaient résistantes aux céphalosporines de première génération (22,6%). Les Gram négatif étaient plus résistants à la ceftazidime qu'aux aminosides: 23,1% versus 7,1%. Les infections fungiques répondaient selon la littérature à un traitement par les azolés. Selon les études multicentriques de l'IPPR, des variations régionales significatives de sensibilité des germes ont été décrites (Tableau 3). Cette variabilité dans la sensibilité des antibiotiques est probablement due à la divergence des choix d'antibiotiques dans différents centres, qui à leur tour sont influencés par l'expérience des sociétés savantes à l’échelle de chaque pays, les recommandations, les considérations économiques et les problèmes de disponibilité de certaines molécules [9]. Ces données soutiennent les recommandations de l'IPPR concernant l'antibiothérapie empirique de la PI qui doit tenir compte de l'histoire du patient, de l’écosystème bactérien et la sensibilité aux antibiotiques de chaque centre [19, 20]. La résistance aux antibiotiques doit être surveillée régulièrement, et le protocole de traitement doit couvrir tous les agents pathogènes graves propres à chaque centre. Les points forts de notre étude étaient le grand nombre de patients inclus et le recul de 10 ans. Notre étude présente les limitations inhérentes de toute étude rétrospective. Aussi, nous n'avons pas pu tracer le profil de portage du *Staphylococcus Aureus* de tous les patients. D'autres études prospectives sont nécessaires afin d’établir un protocole de soins de l'orifice du cathéter (molécules, fréquence) et le profit de thérapies topiques chez les enfants. L’établissement de protocoles de traitement des infections de l'orifice de sortie et des tunellites spécifiques à l'enfant est également primordial avant que l'infection ne diffuse au péritoine. D'autres études sont également nécessaires pour fixer la période optimale de traitement pour les patients pédiatriques ayant une PI afin d'assurer la guérison, d’éviter la sélection de souches résistantes surtout parmi les Gram négatif et d’éviter les complications en particulier l’échec définitif de la DP.

**Tableau 3:** Sensibilité des germes aux antibiotiques dans les différentes études

	USA [9]	Argentine [9]	Europe de L'Est [22]	Europe de L'Ouest [22]	Turquie [2]	Notre série
**Cefazolin**						
**Tous les germes**	10(30%)	4(36%)	20(80%)	37(60%)	33(55%)	25(44%)
**Gram positif**	7(50%)	4(100%)	15(94%)	35(70%)	15(94%)	21(75%)
**Gram négatif**	3(16%)	0(0%)	4(57%)	2(17%)	4(57%)	4(44,4%)
**Ceftazidime**						
**Tous les germes**	18(82%)	9(75%)	18(72%)	32(67%)	33(65%)	Non testé
**Gram positif**	2(67%)	1(100%)	6(55%)	12(50%)	10(50%)	Non testé
**Gram négatif**	16(84%)	8(73%)	11(92%)	20(83%)	23(77%)	8(61,5%)
**Aminosides**						
**Tous les germes**	38(79%)	14(88%)	25(69%)	83(89%)	55(82%)	24(77%)
**Gram positif**	12(67%)	2(100%)	15(63%)	56(86%)	29(81%)	15(82%)
**Gram négatif**	26(87%)	12(86%)	9(82%)	27(96%)	26(87%)	9(72%)
**Glycopeptides**						
**Gram positif**	23(100%)	6(100%)	24(96%)	72(96%)	48(98%)	49(100%)

## Conclusion

Les résultats obtenus montrent que dans la majorité des cas il s'agit de bactéries communautaires, ce qui explique que les échecs thérapeutiques sont rares, même en cas de négativité de la culture du LDP. L'antibiothérapie empirique devra couvrir les germes à Gram positif par la vancomycine et les germes à Gram négatif par la ceftazidime. Les aminosides seront réservés en seconde intention aux germes résistants.
